# Considerations for Measurement of Embryonic Organ Growth

**DOI:** 10.1002/ar.23908

**Published:** 2018-10-05

**Authors:** Stuti Prakash, Bouke A. de Boer, Jaco Hagoort, Quinn D. Gunst, Jan M. Ruijter, Maurice J. B. van den Hoff

**Affiliations:** ^1^ Department of Medical Biology Amsterdam University Medical Centers, Academic Medical Center Amsterdam The Netherlands

**Keywords:** 3D‐reconstruction, alignment, cell size, immunocytochemistry, morphometry, tissue growth

## Abstract

Organogenesis is a complex coordinated process of cell proliferation, growth, migration, and apoptosis. Differential growth rates, particularly during cardiogenesis, play a role in establishing morphology. Studies using stereological and cell sorting methods derive averages of morphogenetic parameters for an organ. To understand tissue composition and differential growth, the researcher must determine a number of morphogenetic parameters in the developing organ. Such measurements require sectioning to enable identification of organ borders, tissue components and cell types, three‐dimensional (3D)‐reconstruction of sections to visualize morphology and a 3D‐measurement scheme to build local morphogenetic information. Although thick the section confocal microscopy partially solves these issues, information loss at the section surface hampers the reconstruction of 3D morphology. Episcopic imaging provides the correct morphology but lacks histological procedures to identify multiple cell types. The 3D‐measurement scheme is based on systematic sampling, with overlapping sample volumes, of the entire organ in the aligned image stack. For each sample volume, morphogenetic variables are calculated and results projected back to the cube (boxel) at the sample volume center. Boxel size determines spatial resolution of the final quantitative 3D‐reconstruction whereas size of the sample volume determines the precision of the morphogenetic information. The methods described here can be used to measure tissue volume, proliferation and cell size, to determine contribution and distribution of cell types in a tissue and to display this information in a quantitative 3D‐reconstruction. Anat Rec, 302:49–57, 2019. © 2018 Wiley Periodicals, Inc.

Growth, the increase in tissue volume, can be defined quantitatively in terms of increase in the number of cells or increase in the volume of cells or a combination of both. Therefore, measurements must be performed at both the tissue and cellular levels to determine the mode and extend of organ or tissue growth. Moreover, different cell types that make up the tissue may proliferate and increase in volume in a cell type specific way and thus contribute differentially and specifically to the growth of the organ as a whole. These different contributions may qualitatively and quantitatively change with ongoing development. Development of the heart is an excellent example in which researchers constantly struggle with the changing three‐dimensional (3D) morphology during development and the contribution of extracardiac cell populations. In short (for review see Sylva et al., 2014), cardiac development starts with the differentiation of precardiac mesodermal cells into cardiomyocytes and endocardial cells while forming a linear tube. This linear heart tube elongates by the addition of cardiomyocytes that differentiate from progenitor cells located at both its anterior and posterior border. While the length of the heart tube increases the tube loops to the right. During looping the cardiomyocytes located at the outer curvature are induced to differentiate and proliferate to form the ventricular chambers. Subsequently, the formation of the four chambered heart is completed by the formation of septa and valves. During this entire developmental process extracardiac cells from the proepicardium and cardiac neural crest are added, which contribute the fibroblasts, coronary vasculature and neural innervation. Taken together, not only the 3D shape of the forming heart but also the numbers and relative contribution of cardiomyocytes and non‐myocytes, their volume and their proliferation rate continuously change. In this manuscript we describe a method to prepare quantitative 3D‐reconstructions which show the values of morphogenetic variables in the 3D context of the developing heart. This procedure can thus be used to visualize local differences in growth and composition and provide detailed insights into morphogenetic processes (de Boer et al., 2011). The qualitative 3D‐reconstructions can also be used to study patterns of (co‐)expression of proteins or mRNAs, genetic labels in genetically modified organisms and morphological changes in any tissue or organ of interest (Soufan et al., 2003; Soufan et al., 2007).

When studying embryonic development, the preservation of the 3D‐geometry of the organs and their relations is essential. Therefore, to study parts of small embryonic organs, sectioning of the tissue and studying the individual sections was, and still is, the technique of choice (Ruijter et al., 2004). In the past, it was required to mentally or artistically reconstruct these two‐dimensional (2D)‐images to obtain an insight into 3D‐morphology; a subjective exercise with the risk of bias being introduced by the researcher or artist. With specialized software it is now possible to prepare an unbiased 3D‐reconstruction from a stack of sections. The resolution of the information collected using this method is determined by the section thickness and the properties of microscope and camera. When these are properly chosen, 3D‐reconstructions can be prepared with a resolution at the cellular level (Chieco et al., 2013). Although the original 3D‐structure of the organ can be faithfully restored, the resolution of such a reconstruction is too high for biological interpretation of the morphogenetic information. This is because the continuous trends in morphogenesis are detected as binary information: cells are either positive or negative for the specific staining and the continuous trends remain hidden in a cloud of variously colored particles. Values of morphogenetic variables (e.g., cell density and cell size) of the whole organ or of a complete tissue‐of‐interest can be determined with unbiased stereological and morphometric procedures (Howard and Reed, 1998) but such an approach is unsuitable for obtaining morphogenetic information for each location in the 3D‐structure. Alternatively, one could consider confocal microscopy on thick sections or episcopic block surface imaging. However, neither will result in satisfactory results. Using confocal microscopy, the information loss at the surface of the thick section will hamper the accurate reconstruction of the complete 3D morphology (Megason and Fraser, 2003). Though the 3D‐morphology obtained using episcopic block surface imaging is superior, the technique currently misses the versality of histological procedures to identify multiple cell types (Weninger and Mohun, 2002). The best compromise is a 3D quantitative reconstruction using planar measurements and profile counts that have to be applied according to a 3D‐measurement scheme (see also for review: Ruijter et al., 2004). The quantitative 3D‐reconstruction protocol presented here consists of two complementary methods (Fig. 1): a qualitative 3D‐reconstruction of the morphology and a quantitative 3D‐measuring scheme resulting in the local values of, for example, labeling index and cell size (Soufan et al., 2007). The qualitative part of the method identifies and describes the organ or tissue‐of‐interest and results in a surface reconstruction. The quantitative part depends on specific staining to identify individual nuclei, specific cell types and structures of interest (Fig. 2). The 3D‐measurement procedure then provides local quantitative information which can be mapped onto the morphological surface reconstruction. The combined result is a quantitative 3D‐reconstruction showing quantitative trends in morphogenetic data in the correct 3D morphological context (Soufan et al., 2006; de Boer et al., 2012).

**Figure 1:**
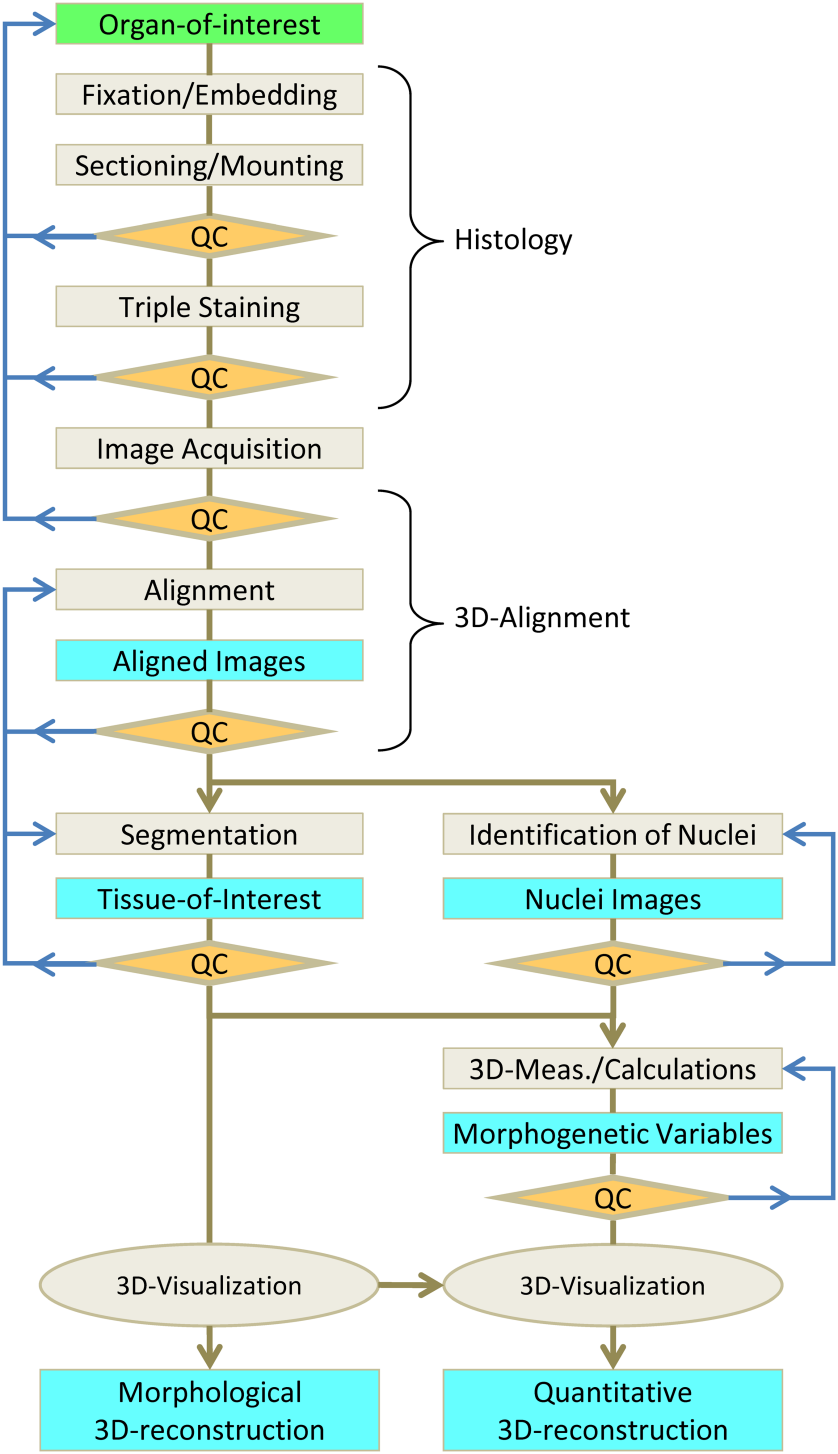
Quantitative 3D‐reconstruction Methodology. This flowchart illustrates the protocol of the qualitative and quantitative 3D‐reconstruction procedure. It starts with basic histology including embedding and sectioning of the organ‐of‐interest followed by image acquisition and 3D‐alignment. The procedure then splits into a morphological path (left) and a quantitative path (right). The former path results in the tissue‐of‐interest achieved through segmentation which then leads to 3D‐visualization of the morphology. The latter path includes identification of nuclei and a local 3D‐measurement protocol and results in 3D‐visualization of the quantitative data. Light‐gray boxes indicate processing steps, of which the main results are indicated by cyan boxes. The orange diamonds represent the quality control (QC) steps in the protocol that may lead to repeating a series of steps (blue arrows).

**Figure 2:**
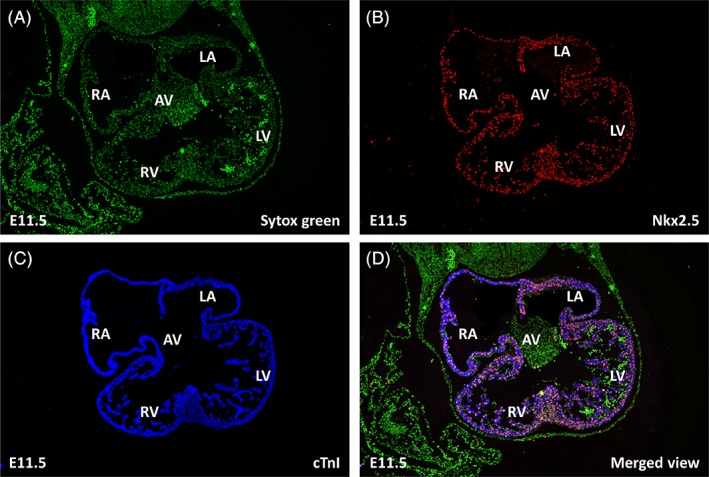
Images of a triple‐stained section of mouse heart tissue at embryonic day 11.5. The images show the three different staining procedures done on each heart section. Panel **A** shows nuclear Sytox green staining. Panels **B** and **C** show immunofluorescent staining with antibodies against Nkx2.5 and cTnI, respectively. In panel **D** the above three staining results are merged. Abbreviations: AVC, atrioventricular canal; LA, left atrium; LV, left ventricle; RA, right atrium; RV, right ventricle

In short, the 3D‐measurement procedure involves sectioning and mounting, staining, image acquisition, 3D‐reconstruction, measurement, calculations, and display. For each of these main steps of this quantitative 3D‐reconstruction procedure the important considerations will be described and discussed. Quality checks should be implemented in each of the histological, 3D‐reconstruction and 3D‐measurement procedures to ensure the quality of the 3D‐reconstructions and the validity of the morphogenetic information. This means that at specific points in the procedure a choice has to be made to either continue with the current data or to discard the data and start over again at a specific earlier step in the procedure or, in the most severe case, start with a new specimen.

## Definitions

**Figure 3:**
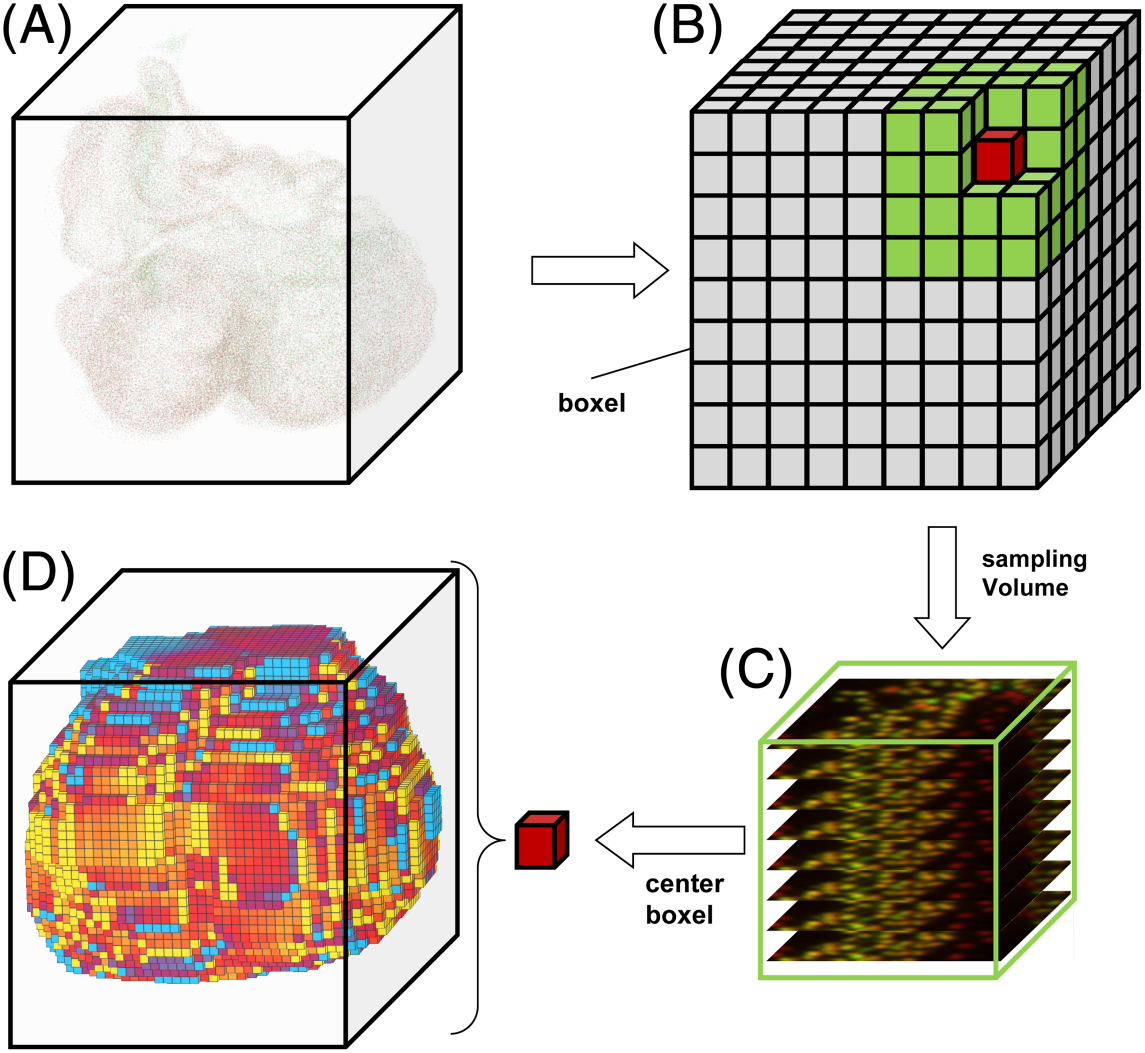
Illustration of the 3D measurement procedure. Panel **A** shows the identified nuclei as a 3D cloud of the aligned stack of images and its bounding box. This volume of the bounding box is subdivided into a 3D‐matrix of boxels with a linear dimension of 26 μm (not to scale) and the sample volume (green, 130 μm) (**B**). Each sample volume includes square regions of several cross sections of the tissue‐of‐interest (**C**). 3D‐measurements are performed by sliding the sample volume (green) over each *X*, *Y*, and *Z* position, performing the measurements and projecting the morphogenetic results into the center boxel of each sample volume resulting in a 3D matrix of morphogenetic values (**D**)

The pixel values in 2D‐gray scale images range from low (generally displayed as black) to high (white). When section thickness is taken into account, the individual 2D‐pixels display the information contained in the 3D‐voxels of which the pixels are projections. The pixel value range occurring in unstained parts of the tissue is defined as background whereas foreground, or signal, is found in the specifically stained tissue components. Foreground and background areas both represent cross sections through 3D‐structures. Discrete 3D‐particles, for example, cell nuclei are represented by profiles in 2D‐images (Fig. 2). In the 3D‐measurement scheme the number of (specific) nuclear profiles and the (specific) tissue area are measured in a stack of square measurement fields which together form a 3D‐measurement volume, or sample volume, spanning several sections (Fig. 3). The measurement results are then used to calculate the number of nuclei per tissue volume from which local cell size and labeling indices are derived. These morphogenetic variables are projected into a cubic volume, dubbed “boxel”, at the center of the sample volume.

## Considerations for Generating a 3D‐Reconstruction

Below we will illustrate and discuss the individual steps and considerations when preparing a quantitative 3D reconstruction of the number of cardiomyocytes in a mouse heart of embryonic day 11.5. However, this procedure and the considerations hold for hearts of other developmental stages and even for other organs.

### *Sectioning and mounting*

Appropriate fixation and embedding of tissue is required to preserve tissue structure, to enable serial sectioning and perform the required staining of the sections. The thickness of the sections depends on the planned staining procedure (Moorman et al., 2000). In general, 7‐μm thick sections are used for immunohistochemistry and 12‐μm sections for *in situ* hybridization. It should, however, be taken into account that in thicker sections it is difficult to distinguish individual nuclear profiles because the microscope image shows a projection of the section. Because the diameter of a https://en.wikipedia.org/wiki/Mammal nucleus is ~6 μm and comprises on average 10% of the total volume of the cell (Alberts, 2014), profiles of individual nuclei can and will overlap in these sections.

Sectioning paraffin‐embedded tissue results in compressed sections that need to be stretched by placing the sections on warm water. The time and temperature, at which the stretching of the sections is done, are two crucial factors in the 3D‐reconstruction procedure and have to be set precisely and monitored critically to avoid over‐ as well as under‐stretching of the sections. Fixed stretching temperature and duration guarantee minimal variation and result in a well‐aligned 3D‐reconstruction and thus consistent 3D‐measurements.

### *Quality control*

When a significant number of sections are lost during the sectioning and/or mounting procedure the entire series of sections of the specimen should be discarded and the entire procedure should be repeated. For large organs, and thus large series of sections, substitution of a lost section by an image of a neighboring section can be considered. This should never be done for more than one section in a row. Nor should there be more than one substitution in a sample volume to avoid bias in morphometry.

## Specific Immunohistochemical Staining

The basic staining procedure that can be used to generate quantitative 3D reconstructions is a double immunofluorescent staining procedure with additional fluorescent staining of all nuclei (Fig. 2). However, note that the 3D‐measurement procedures can be applied to any consecutive series of sections on which all nuclei, the nuclei of a specific cell type and a specific tissue component can be distinguished microscopically.

### *Unmasking epitopes (antigen retrieval)*

Heating of mounted sections is done to unmask epitopes or retrieve antigens shielded due to the fixation. Unmasking thus allows recognition by, and binding of, the antibodies. We found that most antibodies that require such antigen retrieval at a late developmental stage do not need this treatment when the specimens are younger than embryonic day (E) 11.5. Deformation as a result of the heating of the section is negligible. However, one should carefully check whether no parts of the tissue were lost during heating.

### *Non‐ionic detergent treatment*

The most frequently used detergents of this family are Triton‐X100, Nonidet‐P40, or Tween‐20. These detergents dissolve membranes and thus enable antibodies to penetrate the tissue more easily. However, the need for detergent treatment depends on the antibody and the developmental stage of the embryo. Most antibodies that require detergent treatment to successfully stain embryos at late developmental stages do not require such treatment when sections are taken from embryos of E12.5 or younger. When detergent treatment is essential for young embryos the detergent concentration should be carefully titrated to avoid dissolving the tissue.

### *Incubation*

To reduce the amount of antibody required, incubations are performed in droplets covering the sections with slides lying flat. A hydrophobic barrier surrounding the section is applied with a PaP pen (Immunotech, Beckman Coulter) to keep the droplets of incubation medium in place. It is essential to prevent evaporation of the medium by placing the slides in a humidified closed box. Washing steps in between incubation steps should be done in large volumes and are most easily and efficiently performed with the slides placed in a rack, allowing easy repetitions.

### *Antibody dilution*

The dilution factor of an antibody depends on the specific antibody and should be determined in a pilot experiment. The optimal antibody concentration is the dilution which shows the strongest specific staining intensity in combination with the lowest background staining. To increase this difference one can consider increasing the salt concentration (up to 1.5 M NaCl) or decreasing the incubation temperature down to 4°C. To allow comparison between positive and negative tissue compartments, it is essential that the sections used for testing dilutions comprise both tissue compartments.

### *Fluorescence bleaching*

All incubation steps using fluorescent dyes have to be performed in the dark to protect the dyes from photobleaching. To avoid photobleaching the stained sections should be stored in the dark at 4°C and the number of excitation and emission cycles should be minimized. Further reduction of the fluorescence as a result of chemical bleaching of the fluorescent dyes can be achieved by using an anti‐fading mounting medium. Many (expensive) commercial media are available but a cheap alternative is supplementing 1% 1,4‐diazabicyclo[2.2.2]octane (DAPCO) and 50% glycerol to phosphate‐buffered saline pH 7.6. Finally, image acquisition should be performed in a standardized way to avoid bleaching differences between sections.

### *Quality control*

If the specificity and/or intensity of the staining are not good enough to enable efficient identification of cell types, tissues or organ compartments, the entire series of the sections should be discarded and the staining should be repeated on newly fixed or sectioned tissue.

## Image Acquisition

Images of each of the sections containing part of the organ are acquired using a microscope and camera setup (Chieco et al., 2013). The microscope setup used in this study includes an upright fluorescence Leica DM6000 microscope, with a CTR6000 Control Unit, an EL6000 Light Source, and objectives: PLAN FLUOTAR 5×/0.15, PLAN APO 10×/0.40, PLAN APO 20×/0.70, attached to a Dell workstation and two 20″ monitor screens. Achromatic (PLAN) lenses need to be used to avoid, or at least reduce, chromatic and spherical aberration, because fluorophores emit light at different wavelengths. The used camera is a Retiga EXi Fast 1394 (Qimaging) camera with the following specifications: 1.4 MPixel, 12‐bit monochrome, cooled CCD, and photo element size of 6.45 × 6.45 μm. For image acquisition Image Pro Plus MDA (MediaCybernetics) and the advanced fluorescence acquisition module are used. For the imaging of large sections, the setup is equipped with a Marzhauser XY scanning stage and Oasis‐Blue controller (Objective Imaging). The system is calibrated to ensure that the camera is properly aligned with the microscope and scanning stage. This system can be used to acquire images of series of fluorescent and non‐fluorescent sections. Issues to consider before and during image acquisition are the following.

### *Image magnification*

Image magnification should be determined to enable faithful visualization of the 3D‐reconstruction and valid determination of tissue area, tissue volume and cell size. To this end, each image series should be accompanied by an image of a microscopic ruler which can then be used to determine the spatial resolution or actual pixel size (μm of tissue per pixel) of the image (Chieco et al., 2013). Note that for reliable detection of profiles of nuclei one requires nuclear profiles to occupy at least 6 × 6 pixels. In our setup, we meet this requirement with a 10× objective in combination with the 1.4 megapixel camera, resulting in an image resolution of ~0.9 μm/pixel.

### *Fluorescence filter sets*

The choice of the filter set is important for the recorded fluorescence to be specific for the applied staining. In a fluorescence microscope a filter set comprises three components: an excitation filter, a dichroic and an emission filter. The fluorescence filter set should be carefully selected to allow the recording of the specific excitation of the interrogated fluorophore only. Bleed‐through of other fluorophores present in the same section should be carefully accessed, to avoid over‐projection or identifying false positives. In Table 1, a combination of filter sets is provided that allows discrimination of five fluorescent dyes in one section. Other filter sets can be designed and checked with freely available web‐based tools, like the Fluorescence Spectra Viewer (https://www.thermofisher.com).

**Table 1:** Fluorescent filter sets

Filter	Fluorescent dye	Excitation filter	Emmision filter
1	Alexa 405	387/11	438/24
2	Sytox Blue	434/17	475/20
3	Alexa 488	485/20	527/20
4	Alexa 555	545/30	610/75
5	Alexa 647	620/60	700/75

The table lists the fluorescent dye, excitation wavelength and emission wavelength of the filter sets used for the acquisition of the images.

### *Large sections*

When the tissue area is larger than the field of view, you have to use tiling and stitching software, like the IPP Stage Pro module (MediaCybernetics), to acquire sets of overlapping images. When tiling and stitching is performed, the *X*–*Y* movement of the scanning stage needs to be calibrated with the *X*–*Y* matrix of the CCD camera. The overlapping regions are then cropped off by the software. For easy acquisition of large image sets (many sections and slides) custom made macros can be used. Especially when tiling and stitching is applied, chromatic aberration should be evaluated when a large difference between the wave lengths of the emitted light of the used fluorophores is present and when also the periphery of the lens is used.

### *Systematic and consistent organization of image files*

In our experience, storage of the image files that are generated in the course of a quantitative 3D‐reconstruction requires a folder structure that starts with a main folder identifying the original specimen and then forms a tree structure that parallels the generation of different types of images in the quantitative 3D reconstruction process. Such a folder structure provides easy systematic access to the original images as well as the aligned, segmented, and 3D‐Tiff images for each of the fluorescence channels that are recorded. This folder structure helps to keep track of the images, and thus enables the researcher to back‐track in case the quality check indicates that some steps in the procedure should be redone.

## 3D‐Image Analysis and Reconstruction

To reconstruct the 3D morphology of the original organ and the distribution of cell types in this organ, the 2D images of the sections have to be processed into a 3D‐reconstruction and the different cell types have to be identified in these sections. The 3D image analysis and reconstruction procedures thus consist of 3D‐alignment, segmentation and identification of specifically stained nuclei (Fig. 1).

## *3D‐Alignment*

To create a 3D‐reconstruction from sections, a dedicated software package, like Amira (version 5.4.3, http://www.amira.com), has to be used. Alignment of the images, acquired from the stained sections, should be done on as many tissues or organs as possible, not just the organ‐of‐interest. Most often alignment can be done on the image showing the background staining or the auto‐fluorescence of the tissue. The 3D‐reconstruction software performs an iterative procedure to align pairs of images. A narrow range of gray value within the tissue background often suffices to obtain a satisfactory result. When the 3D alignment is optimized and of sufficient quality, the alignment parameters can be saved and applied to the images of the other channels, because these channels were recorded without moving the microscope stage.

### *3D‐connectivity*

It might not be possible to reach 3D‐connectivity for all structures. For example, thin‐walled atria in the embryonic heart, which have a wall thickness close to the section thickness, will always appear as discontinuous structures. Such discontinuities do not affect the 3D‐measurement procedure. However, for 3D‐visualization it might be required to improve the connectivity by interpolation to obtain a better surface view of the 3D‐reconstruction. Note that the 3D measurements should always be performed on the original images.

### *Image conversions*

Most 3D‐reconstruction software is only compatible with 8‐bit (gray value range 0–255) or 16‐bit (gray value range 0–65,535) images. When the camera does not generate images in these formats, image conversion has to be performed. In case the camera produces 12‐bit (gray value range 0–4,095) images, these have to be converted to 16‐bit images without stretching, to avoid change or loss of information. In the resulting 16‐bit image only the first 4,096 gray values are used; the unused gray values can be ignored in the display of the images in the 3D‐reconstruction program.

### *Image numbers*

Frequently the software can only handle consecutively numbered images. This may require that the researcher renumbers the stack of images for each channel. For easy renumbering of images freely available programs can be found online, for example, Bulk Rename Utility (http://www.bulkrenameutility.co.uk).

### *Quality control*

When images are automatically aligned, the researcher needs to visually inspect the result. This can be facilitated by showing an aligned image pair in complementary colors (e.g., red and green); the overlapping structures in the two consecutive images are then shown in white. This inspection and correction is most efficiently done by starting with the middle section pair of the image stack and working both directions. When too many artifacts due to tissue compression are identified, the image set should be discarded and one should start with sectioning a new specimen.

## Segmentation of Tissue‐of‐Interest

For the 3D‐measurement procedure, the tissue‐of‐interest should be identified in the images of the channel in which this tissue is specifically stained. This process of dividing an image into different regions (also called segments or labels) is referred to as image segmentation. Segmentation effectively converts the grayscale images into binary images in which the tissue (or stained structure) of interest (foreground) is distinguished from the remaining tissue and glass (background).

In a stack of aligned images the specific signal of stained tissue (e.g., myocardium) can be isolated from the background using a threshold‐mode in the 3D‐reconstruction software. Since the staining intensity may vary between sections, it may be necessary to set this threshold manually per image: the selected area is often shown in a contrasting color and should match the area of the stained tissue‐of‐interest. When a threshold is automatically set a quality control per image is needed. If no specific staining is available, one can manually trace the tissue‐of‐interest. This is not preferred as it is laborious and prone to bias introduced by the researcher.

### *Quality control*

Differences in staining intensity between sections may severely hamper the consistency of the segmentation. When many images require adjustments of the segmentation threshold or when manual tracing of the tissue‐of‐interest is required it is better to discard the images and restart the staining procedure.

## Identification of Nuclei

The 3D‐measurement protocol it based on the identification and counting of nuclear profiles to allow the calculation of labeling index or cell density. The identification of all nuclear profiles in histologically stained sections is often hampered by gradients in staining intensity that inevitably occur. These staining gradients can be removed by subtracting a low pass filtered image (in our case a 10 × 10 pixel filter) from the original image resulting in a local‐maxima image. In this local‐maxima image a threshold can then be set to select the nuclear profiles. The threshold depends on the size of the kernel used in the low pass filter which needs to be set such that in the image the majority of the objects will be single nuclear profiles. In some cases, especially when nuclei are densely packed and the sections are relatively thick, nuclear profiles will overlap. In that case, image analysis functions to split such clusters of profiles, based on the curvature of their boundaries or the pixel value profile of their interior, are available in most image analysis packages. The 3D‐Measurement Toolbox applies such a function and divides profiles in two when the profile area is larger than twice the median profile area (de Boer et al., 2012).

The identified nuclei then serve as a mask to locate nuclei in the image of the channel that shows the specifically stained nuclei. It is essential that the alignment of the images is corrected at the resolution of the individual nuclei. In general, positive nuclei are identified as those specifically stained nuclear profiles for which the staining intensity in the nuclear profile is significantly above the local background staining in that image (de Boer et al., 2012).

### *Quality control*

It is essential to check whether all nuclei have been identified and overlaid correctly with the specifically stained nuclei. This can be done by comparing two images side by side. However, it is more efficiently carried out when a control image is generated. In such a control image the positive and negative nuclei are identified with different colored circles. Missed, or wrongly assigned, nuclei are rapidly recognized in these images. When not all nuclear profiles are correctly identified the size of the kernel in the low pass filter, the threshold set in the local maxima image and/or the criterion for declaring a profile to be positive should be adjusted. An additional point of attention at this point is the effect of chromatic aberration. Especially in the periphery of the images chromatic aberration can cause a problem. The researcher should make sure that the generically stained nuclei overlap with the specifically stained nuclei. If chromatic aberration is causing a problem, there are three practical solutions to solve the issue. The researcher can use only the center of the image, inevitably leading to more tiles per section. Alternatively, the experiment needs to be redone and other fluorophores have to be chosen of which the emission wavelengths are closer to each other. The most expensive solution is to buy a new lens which is corrected for chromatic aberration in the range of the wavelengths used.

## 3D‐Measurement

In practice, 3D‐measurement of morphometric variables is based on the division of the tissue‐of‐interest into a 3D‐matrix of virtual cubes, called “boxels” (Fig. 3). One boxel consists of multiple voxels and can span multiple sections. The variables are then measured in a sliding 3D‐window which is a cube‐shaped sample volume that consists of one, eight (2 × 2 × 2), 27 (3 × 3 × 3), and so on boxels. After each measurement, the resulting values of morphogenetic variables in this sample volume are assigned to a boxel in the center of the sample volume. The sample volume is then moved one boxel in *X*, *Y*, or *Z* direction until the whole volume of the aligned image stack is covered.

### *Boxel volume*

The size of the boxel is mostly chosen in the order of the size of the cells in the tissue (linear dimension of ~25 μm) and is then determined as a multiple of the section thickness.

### *Sample volume*

The size of the sample volume depends firstly on the required number of profiles that has to be counted to reach sufficiently precise results and secondly on the desired spatial resolution of the morphogenetic information displayed in the quantitative 3D‐reconstruction. The required size of the sample volume is especially important for a valid estimation of the labeling index. Given the ever‐present biological variation of about 30%, a labeling index of 0.5 ± 0.075 (mean and 95% confidence interval) was considered to be sufficiently precise. Such a precision can be reached when 171 profiles are counted per sample volume (Soufan et al., 2007; Chieco et al., 2013). This required number of profiles determines the optimal size of the sample volume. Because a larger sample volume decreases the spatial resolution of the quantitative information displayed in the resulting 3D‐reconstruction the researcher may have to accept less precision in favor of spatial resolution. To determine the optimal size of the sample volume, a pilot measurement with stepwise increasing sample volumes around the center boxel has to be performed. After performing the 3D‐measurements the cumulative distribution of the number of profiles counted per sample volume has to be constructed. This graph enables the researcher to choose the required sample volume and to determine the fraction of the tissue volume that is measured with less confidence (Soufan et al., 2007).

### *Measurements*

The following measurements are performed in the parts of the sections that are included in each sample volume: (1) the area of the tissue‐of‐interest, (2) the total number of profiles of nuclei, and (3) the number of profiles of the specifically stained cell type. Nuclear profiles are only counted within the tissue of interest within the sample volume. The values of morphogenetic variables that are measured for each sample volume, are converted into gray values and projected to the center boxel of the sample volume in the image stack. For each variable, this results in a 3D‐Tiff image in which the stack of images is represented as a 3D‐matrix of pixel values (de Boer et al., 2012).

### *Quality control*

To check whether the number of profiles counted per sample volume is sufficient to reach the required precision of the labeling index, the results have to be plotted in a 3D reconstruction. When a scattered pattern is obtained, this indicates that many local values are based on less than the required number of profiles. In that case, the sample size has to be increased and the measurements have to be repeated. When the sample size is too large the 3D reconstruction will not show detailed information.

## Calculation of Local Values of Morphogenetic Variables

For the calculation of morphogenetic variables, like cell size or labeling index, another two parameters, fixed per image stack, are required: the section thickness and the mean diameter of nuclei. Both are required to calculate the cell density from the observed number of nuclear profiles per section area. This mean nuclear diameter has to be determined in a separate experiment, for example, with confocal microscopy on thick tissue slices. When the nuclear diameter differs for the different types of cells that are to be distinguished, the mean diameter per cell type should be used in the calculations to reach unbiased cell densities and cell sizes per cell type. Although it is best to avoid any bias, one should realize that even a two times increase in nucleus volume, as might occur in dividing cells, has only limited effects on the observed cell size (Chieco et al., 2013). With equations from stereology (Weibel, 1979; Howard and Reed, 1998) the local measurement results are then used to calculate the local values of morphogenetic variables.

### *Number of cells*

The number of nuclei per unit volume (*N*_*V*_) can be derived from the number of profiles per tissue area (*N*_*A*_ *= N*_profiles,total_ */∑*(*A*)), the section thickness (*t*) and mean nuclear diameter (*D*) according to Abercrombie's equation (Abercrombie, 1946): *N*_*V*_ *= N*_*A*_ */*(*D + t*). This equation corrects for the fact that the observed number of nuclear profiles per unit area in a section depends on the number of nuclei per unit volume, the size of the nuclei and the thickness of the section. It therefore also corrects for the fact that some nuclei may be counted twice because their profiles were present in the serial sections in the sample volume. The calculation of number per volume should be performed per cell type. When the diameter of the specifically stained nuclei differs from the diameter of the other nuclei, the respective diameters should be included in the calculation of the number of nuclei per unit volume (*N*_*V*_) per cell type.

### *Cell size and labeling index*

The cell size, defined as the average tissue volume per nucleus, is the inverse of the number of nuclei per unit volume: *Cell size = 1/N*_*V*_. The labeling index (LI) is defined as the fraction of cells that is specifically stained and can be calculated from the number of nuclei per unit volume: *LI = N*_*V*,positive_ */*(*N*_*V*,positive_ + *N*_*V*,negative_).

### *Tissue volume and total cell number*

The tissue volume per sample volume is calculated according to the Cavalieri principle as the section thickness (*t*) times the sum of the observed tissue area (*A*): *V*_tissue_ *= t.∑*(*A*). The number of nuclei in the sample volume can then be calculated by multiplication of the number of nuclei per unit volume and the tissue volume: *N = N*_*V*_. *V*_tissue_ and pooled for the total tissue of interest to determine the total cell number.

## Visualization in a 3D‐Reconstruction

**Figure 4:**
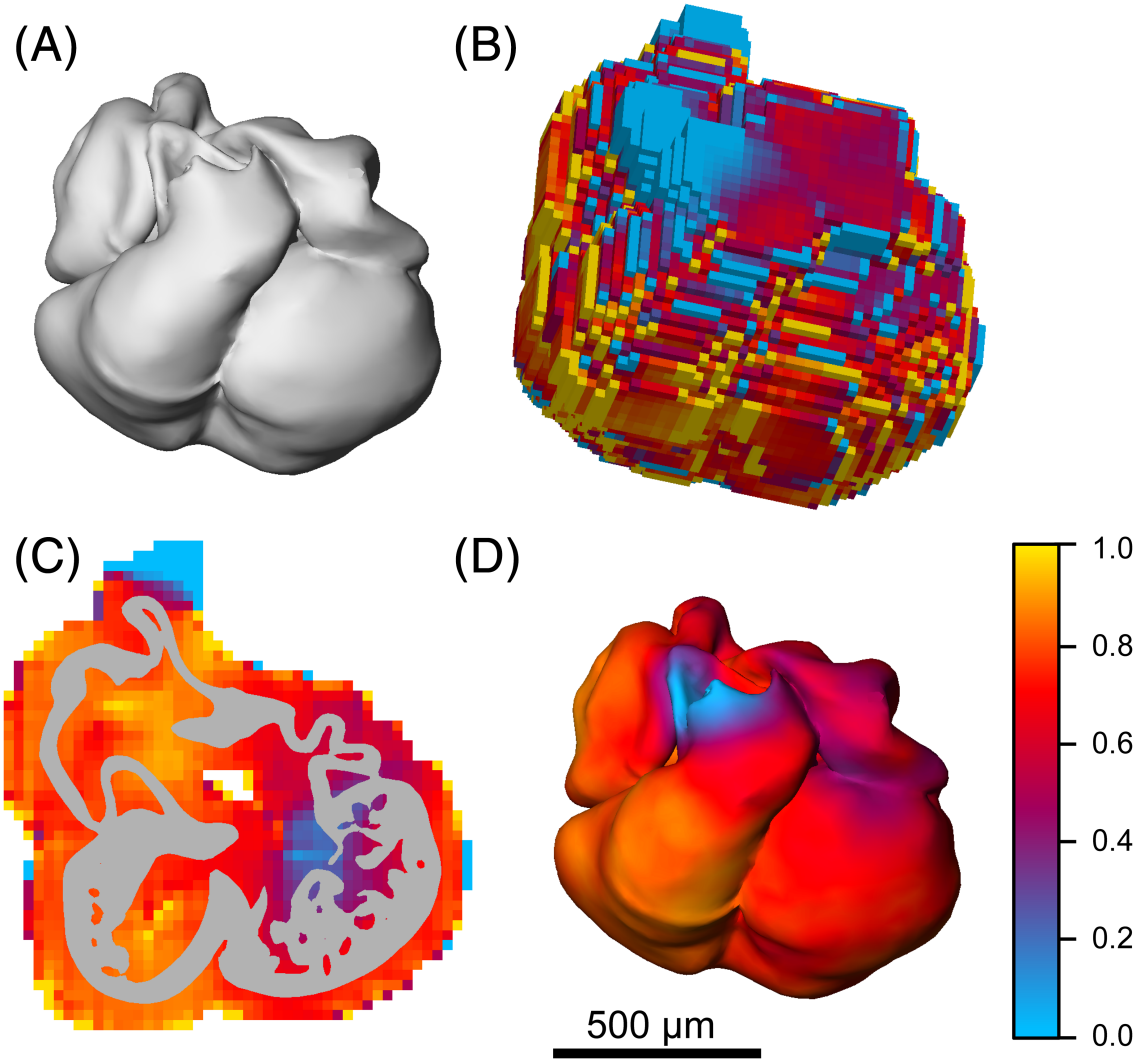
3D‐Visualization of labeling index. Segmentation of the myocardium results in a 3D‐reconstruction of the morphology of the heart at embryonic day 11.5 (**A**) whereas the 3D‐measurements result in a 3D‐matrix of boxels displaying the locally measured labeling index (**B**). Masking of these local labeling indices with the segmented tissue‐of‐interest (**C**) results in the quantitative 3D‐reconstruction displaying the labeling indices in the proper morphological context (**D**). In this example, the fraction of Nkx2.5 labeled cells was used to visualize the cardiomyocyte population in the developing mouse heart (scale bar indicates 500 μm). Also see the 3D‐PDF in the supplemental data for an interactive 3D visualization.

To visualize the measured and calculated morphogenetic variables, the morphological 3D‐resonctruction (Fig. 4A) is combined with each of the 3D‐Tiff files, to which the calculated morphogenetic values are saved (Fig. 4B). The procedure on how to perform this “masking” (Fig. 4C) of the quantitative image set (Fig. 4B) with the morphological 3D reconstruction (Fig. 4A) to obtain the quantitative 3D‐reconstruction (Fig. 4D) depends on the 3D‐reconstruction software used. In general, the procedure can be visualized as shown in Figure 4C which shows one section with the segmented profile of the heart on top of the image from the 3D‐Tiff stack that contains the labeling index information. Note that the quantitative information extends beyond the heart contour because every sample volume that contains the tissue‐of‐interest in included in the 3D‐Tiff. The very high and very low labeling indices at the periphery of the 3D‐Tiff stack and section indicate that these peripheral measurements do not include enough nuclei for reliable estimation of a labeling index. The implementation of this visualization in Amira has previously been described in detail (de Boer et al., 2011). Though Figure 4D only shows a snapshot of the quantitative 3D reconstruction, all data in 3D are available for inspection. In the Figure 3‐D1 we have prepared a representation of this heart which can be inspected from every angle and from the inside, revealing information which is inevitably not visible at the outside of the heart.

## Final Considerations

This article shows that 3D‐measurements can be used to visualize local quantitative information in the 3D‐context of the tissue‐of‐interest. Planning an experiment with 3D‐reconstructions has to take into account that in biological experiments one has to deal with variation between specimens, samples and measurements. In general, the largest contribution to the variation is at the specimen level (20%–30%) and the smallest at the measurement level (<5%; Howard and Reed, 1998). On the other hand, replicate measurements are often less costly than replicate biological specimens. The variation per level, its propagation through the levels and the cost per level can be used to find the optimal design that gives the smallest variation with lowest total cost (Gundersen and Osterby, 1981). Therefore, the general paradigm “do more less well” most effectively decreases variation.

The described 3D‐measurement protocol is not completely free of methodological bias because it assumes spherical nuclei with a constant diameter per type of cell. Unbiased stereological approaches would overcome this drawback. However, application of these unbiased approaches is not possible when morphogenetic variables have to be estimated for every position in the developing heart. Some bias has to be accepted in order to be able to present such local information on tissue growth. Therefore, the user of these methods should be aware of the bias that arises by deviations from the assumption that the populations of nuclei in the studied tissue are fully convex and of similar size. As long as the differences between developmental stages or experimental treatments are sufficiently large, such methodological bias should not discourage the researcher from performing the experiment and doing the 3D‐measurement. The added value of a quantitative 3D‐reconstruction cannot be replaced by observations resulting in one value, albeit unbiased, for the whole tissue‐of‐interest.

## Supporting information


**Figure S1**‐**D1**. A 3‐Dimensional model of the fraction of cardiomyocytes, visualized using Nkx2.5 immunofluorescent positive nuclei, in the ED11.5 mouse heart. To activate the model, click on the center of the image above. The model can then be freely manipulated to inspect the model from each angle. Clicking on the preset views at the right side of the window, activates the model in the large screen that can then be freely manipulated, allowing the assessment of the fraction of cardiomyocytes from the interior side of the heart. The color scale is indicated at the left side of the window, ranging from no cardiomyocytes (blue) to 100% cardiomyocytes (yellow). To open Figure 3‐D1 in a floating window that will remain open throughout the document, after the 3D PDF is activated (click the model to activate), right click (or control‐click) on the model, then click View in Floating Window. The 3D PDF will remain open in a resizable floating window as the user advances through the pages.
