# Supplementary material for: Considerations for Measurement of Embryonic Organ Growth
Source: Anat Rec (Hoboken). 2018 Oct 5;302(1):49–57. doi: 10.1002/ar.23908 (PMC7028151; doi:10.1002/ar.23908)

Cardiomyocyte fraction in ED11.5 mouse heart

views

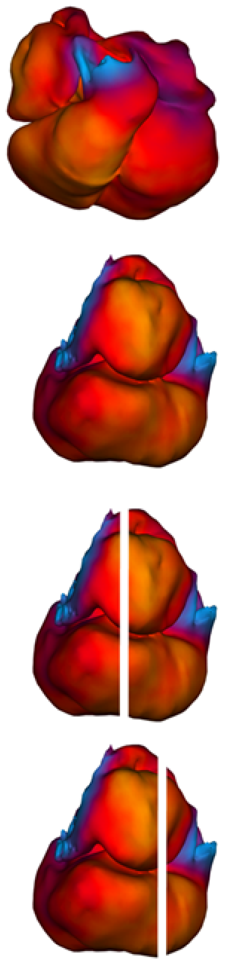

scale

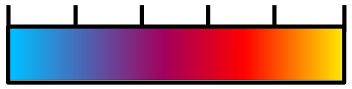

Fraction Nkx2.5 positive nuclei

Department of Medical Biology

http://3d.hfrc.nl

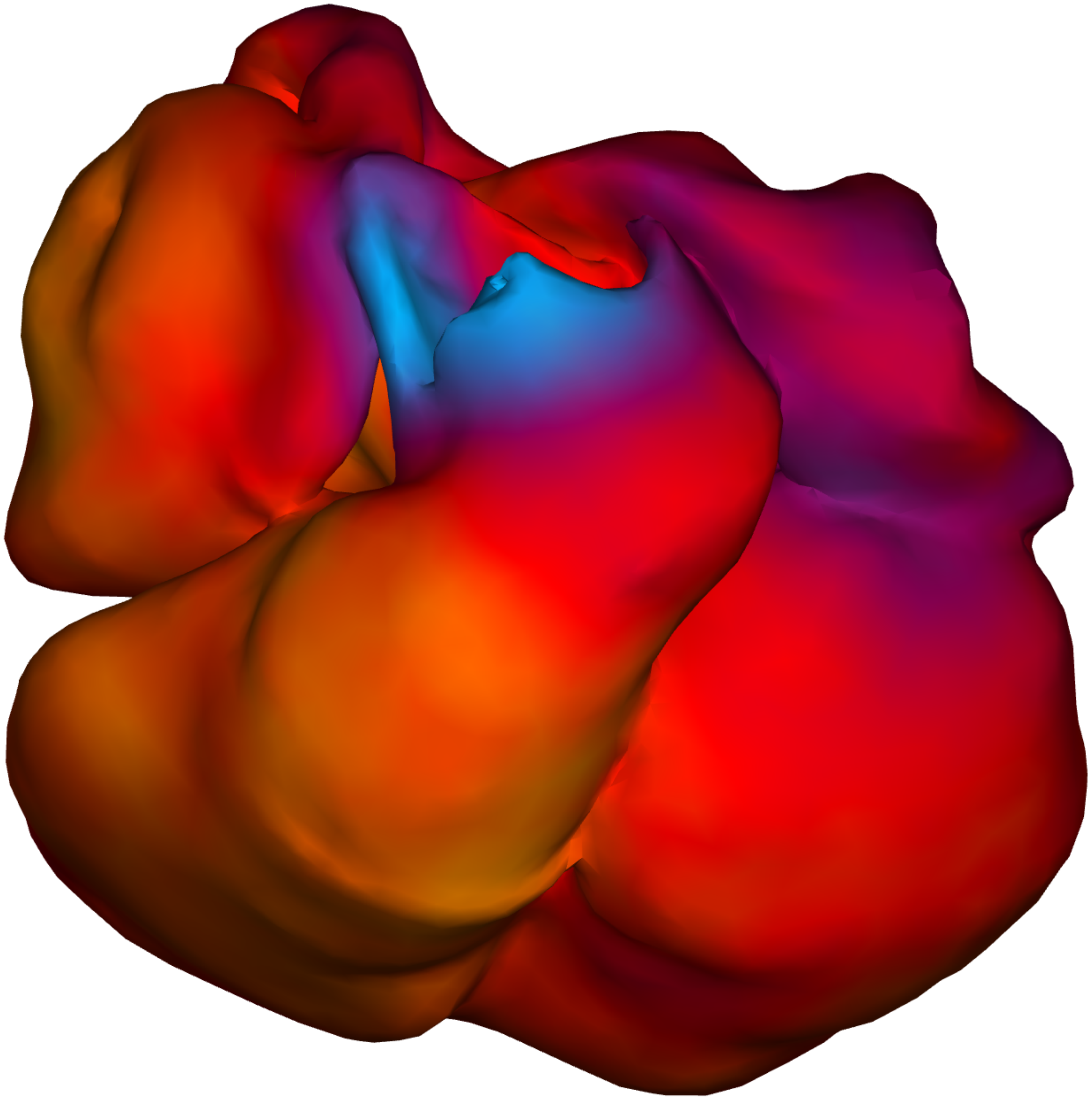

Supplement: Supplementary file 1 — Figure S1‐D1. A 3‐Dimensional model of the fraction of cardiomyocytes, visualized using Nkx2.5 immunofluorescent positive nuclei, in the ED11.5 mouse heart. To activate the model, click on the center of the image above. The model can then be freely manipulated to inspect the model from each angle. Clicking on the preset views at the right side of the window, activates the model in the large screen that can then be freely manipulated, allowing the assessment of the fraction of cardiomyocytes from the interior side of the heart. The color scale is indicated at the left side of the window, ranging from no cardiomyocytes (blue) to 100% cardiomyocytes (yellow). To open Figure 3‐D1 in a floating window that will remain open throughout the document, after the 3D PDF is activated (click the model to activate), right click (or control‐click) on the model, then click View in Floating Window. The 3D PDF will remain open in a resizable floating window as the user advances through the pages. [file AR-302-49-s001.pdf]
